# Altered gut microbiota and metabolites profile are associated with reduced bone metabolism in ethanol‐induced osteoporosis

**DOI:** 10.1111/cpr.13245

**Published:** 2022-06-10

**Authors:** Zhao Liu, Xilin Xu, Yiwei Shen, Yuanyuan Hao, Wenwen Cui, Wenyan Li, Xin Zhang, Hang Lv, Xiaodong Li, Yunlong Hou, Xiaofeng Zhang

**Affiliations:** ^1^ Graduate School Heilongjiang University of Chinese Medicine Harbin China; ^2^ The First Affiliated Hospital of Zhejiang University of Chinese Medicine Hangzhou China; ^3^ The Second Affiliated Hospital of Heilongjiang University of Chinese Medicine Harbin China; ^4^ Key Laboratory of Northern Medicine Base and Application Under Ministry of d Education Harbin China; ^5^ Graduate School Hebei University of Chinese Medicine Shijiazhuang China; ^6^ Shijiazhuang Yiling Pharmaceutical Co., Ltd Shijiazhuang Hebei China; ^7^ College of Integrated Traditional Chinese and Western Medicine Hebei University of Chinese Medicine Shijiazhuang China; ^8^ Heilongjiang Provincial Administration of TCM Harbin China

## Abstract

**Objective:**

Chronic heavy drinking causes ethanol‐induced osteoporosis (EIO). The present study aimed to explore the role of GM in EIO.

**Material and Methods:**

A rat EIO model was established by chronic ethanol intake. Taking the antibiotic application as the matched group of dysbacteriosis, an integrated 16S rRNA sequencing and liquid chromatography–tandem mass spectrometry‐based metabolomics in serum and faeces were applied to explore the association of differential metabolic phenotypes and screen out the candidate metabolites detrimental to ossification. The colon organoids were used to track the source of 5‐HT and the effect of 5‐HT on bone formation was examined *in vitro* *.*

**Results:**

Compared with antibiotics application, ethanol‐gavaged decreased the BMD in rats. We found that both ethanol and antibiotic intake affected the composition of GM, but ethanol intake increased the ratio of *Firmicutes* to *Bacteroidetes*. Elevated serotonin was proved to be positively correlated with the changes of the composition of GM and faecal metabolites and inhibited the proliferation and mineralization of osteogenesis‐related cells. However, the direct secretory promotion of serotonin was absent in the colon organoids exposed to ethanol.

**Conclusion:**

This study demonstrated that ethanol consumption led to osteoporosis and intestinal‐specific dysbacteriosis. Conjoint analysis of the genetic profiles of GM and metabolic phenotypes in serum and faeces allowed us to understand the endogenous metabolite, 5‐HT, as detrimental regulators in the gut‐bone axis to impair bone formation.

## Graphical abstract

A rat ethanol‐induced osteoporosis model was established by long‐term ethanol intake. Taking the antibiotic application as the matched group of dysbacteriosis, an integrated 16S rRNA sequencing and liquid chromatography–tandem mass spectrometry‐based metabolomics in serum and faeces were applied to explore the association of differential metabolic phenotypes and screen out the candidate metabolites detrimental to ossification. The colon organoids were used to track the source of 5‐HT and the effect of 5‐HT on bone formation was examined *in vitro* *.*

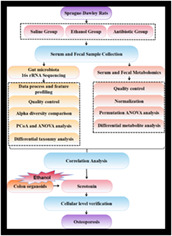

## 1 INTRODUCTION

Chronic heavy ethanol consumption leads to gradual damages to multiple systems,^1^ which represents one of the most common causes of mortality worldwide.^2^ Recent studies have indicated that chronic heavy ethanol consumption can be positively associated with bone impairments and an increased risk for bone fracture.^3^ A large‐scale case control study in Denmark reveals an alcoholism rate of 7.1% in patients with fractures versus only 2.5% in control subjects without fractures.^4^ Although accumulating evidence shows that ethanol is an important risk factor for osteoporosis,^5^, ^6^ it remains largely unknown how bone loss and osteopenia occur as parts of many unwanted consequences of ethanol consumption. It is recently reported that chronic heavy ethanol consumption directly impairs gut microbiota (GM) composition, which might be a pivotal pathogenic factor or aggravate pre‐existing illnesses.^7^ Notably, studies have repeatedly shown that a gut‐bone regulatory axis exists, by which GM composition could indirectly regulate bone metabolism.^8^ However, it remains largely unknown whether the osteoporosis caused by chronic heavy ethanol consumption is related to the impairments of GM composition.

Bone is a dynamic tissue throughout life. The maintenance of healthy bone in human adults depends on the balance of bone resorption and formation, which is called bone remodelling.^9^ The bone loss and osteoporosis following chronic heavy ethanol consumption are mainly due to the imbalance of bone remodelling. This imbalance eventually results in osteopenia, an established risk factor for osteoporosis. Results of human and animal experiments indicate a direct inhibitive effect of ethanol on bone‐forming cells.^10^, ^11^, ^12^ Biochemical and histological evaluation in patients with ethanolic bone disease reveals a marked impairment in bone formation in the face of relatively normal bone resorption. A well‐defined rat model of heavy drinking shows that ethanol‐induced bone loss rests with antiproliferative effects on osteoblasts, ultimately impairing bone remodelling and mineralization.^13^ Although there is increasing evidence that ethanol plays roles in membrane perturbation and transmembrane signal pathways, the specific subcellular mechanisms whereby inhibit cell proliferation are unknown. Notably, Chronic, heavy ethanol consumption has been proved to be detrimental to every tissue of the body, which means, despite the direct impairments, ethanol‐induced bone loss could be secondary by disrupting metabolic homeostasis of hormones, endogenous metabolites, and nutrient absorption, which are critical for bone forming and growth.^14^, ^15^ Several observations have previously suggested that circulating serotonin, as an endogenous metabolite, could be playing a significant role in the regulation of bone mass.^16^, ^17^ The Lrp5 gene deficient mice are shown to have high serotonin levels and a low bone mass phenotype.^16^ While it is still unknown that whether the gut‐derived serotonin levels change in EIO. People with chronic heavy ethanol consumption show lower serum vitamin D levels, indicating mineralization disorders.^13^ A closer examination of these indirect factors may contribute to developing interventional strategies for the treatment of ethanol‐induced bone diseases.

Recent studies, however, reveal that chronic heavy ethanol consumption not only affects the gastrointestinal tract but also induces changes of microbiota composition in the gastrointestinal tract (GIT).^18^ The GM is referred to as the second gene pool of the human body and a resident microorganism, both symbiotic and pathogenic, living in our gastrointestinal tract. Accumulating evidence highlights the importance of GM dysbiosis, which is postulated to be a major factor in human disorders. All clinical and preclinical data suggest that the quantitative and qualitative dysbiotic changes in the GM may contribute to ethanol‐related disorders.^19^ Chronic heavy ethanol consumption may be associated with increased GIT inflammation and intestinal hyperpermeability, resulting in elevated serum levels of lipopolysaccharide (LPS),^20^ systemic inflammation, and eventually tissue damage.^21^ Sjogren et al. for the first time discover the relation between microbiota and bone development and demonstrate the higher bone mass formation in germ‐free mice than that in normal mice.^22^ Subsequently, recent findings, however, provide substantial evidence for the existence of a GM‐bone axis, by which GM influences the skeletal homeostasis via affecting the host metabolism, immune function, and hormone secretion.^23^, ^24^, ^25^ Alterations in the GM may serve as biomarkers or therapeutic targets for glucocorticoid‐induced osteoporosis.^26^, ^27^ It is noteworthy that ethanol‐related disorders are associated with quantitative and qualitative changes of GM dysbiotic, which may contribute to the intestinal hyperpermeability to luminal bacterial products and influence the skeletal homeostasis via the GM‐bone axis. Although such epidemiologic analyses demonstrate the underlying GM‐bone axis mechanism in bone loss, present evidence mainly focuses on the direct damage on the bone‐forming cell by ethanol‐induced oxidative stress, instead of the relevance of GM dysbiosis to bone homeostasis. Hence, it is meaningful to study GM‐related phenotype alterations in ethanol‐induced osteoporosis for understanding the modulating pathways involving in the GM‐bone axis, raising the possibility of therapeutic strategies for ethanol‐induced bone disorders.

In this study, a rat model of chronic heavy ethanol consumption was used to investigate the relevance of ethanol‐induced GM dysbiosis to the metabolic phenotype alterations in the serum and faecal using 16S rRNA gene sequencing and liquid chromatography–tandem mass spectrometry (LC/MS)‐based metabolomics.

## 2 MATERIALS AND METHODS

### 2.1 Animal management and faecal sampling

In this study, a total of 27 adult (7–8 weeks old) male Sprague–Dawley (SD) rats (purchased from Beijing Vital River Laboratory Animal Technology Co.) were used, weighing 250–300 g. Animals were housed in groups of *n* = 3–4 (medium density) rats per cage on a reverse 12 h light/dark cycle under standard temperature and humidity conditioned at the conventional SPF Animal Care Facility. Standard food and tap water were available ad libitum in the home cages. Animals were maintained under constant conditions for 10 days before the start of the experiments and under daily surveillance by veterinary staff and/or experimenters.

All animals were divided into three groups, which including saline (S) group, ethanol (E) group and antibiotic (A) group (*n* = 9 in each group). In ethanol group, rats were gavaged with water containing 20% (vol/vol) ethanol (10 ml/kg, 6 times/week, 1 time/day, 16 weeks). The method was slightly modified based on existing articles.^28^, ^29^ The antibiotic‐treated rats were given antibiotic gavage containing 1 g/L metronidazole and 0.2 g/L ciprofloxacin (10 ml/kg, 6 times/week, 1 time/day, 16 weeks). The rats in the S group were given the same amount of saline by gavage as the E group. To avoid contamination, fresh faeces were collected from the terminal rectum of each rat. A total of 27 faecal samples from the rectum were collected on ice, immediately frozen in liquid nitrogen, and then stored at −80 °C until microbiome and metabolome analysis.

### 2.2 Histological examination

For histological analyses, the colon tissues of rats were dissected and placed whole in 4% formalin fixative overnight. Tissues were then embedded in paraffin and routinely stained with haematoxylin and eosin (H&E), and analysed by a pathologist without prior knowledge of experimental procedures. Histological analysis was performed based on the morphological differences of ethanol in colon injury, including infiltration of granulocytes in the lamina propria mucosae, number of goblet cells, and the thickness of muscularis externa.

For the bone histology and histomorphometry analysis, we used Masson staining. All the fifth lumbar vertebrae were fixed in fixative and decalcified in 10% ethylene‐diaminetetraacetic acid (EDTA, pH 7.0, Genview Biotech, China). The treated tissues were wrapped in paraffin and prepared for paraffin sections (section thickness of 5 μm). After staining, the samples were observed under a light microscope (Olympus, China) to evaluate the change of osteoporosis.

### 2.3 Micro‐computed tomography scanning

All the right tibia bones were dissected, cleaned, and fixed in 4% formalin fixative. Then, they were scanned by micro‐computed tomography (micro‐CT) (SCANCO, Switzerland) with a spatial resolution of 20 μm and the X‐ray tube potential of 90 kV and 480 μA. The tibiae analyses were performed on trabecular bone defined as beginning proximal (a distance of 1% of total bone length) to the growth plate and then extending 10% of total bone length toward the diaphysis, excluding cortical bone. Different microstructural parameters were analysed, which included trabecular bone mineral density (BMD), trabecular bone volume/tissue volume ratio (BV/TV), trabecular number (Tb. N), trabecular bone separation (Tb. Sp) and trabecular bone thickness (Tb. Th). Analyses were carried out by an operator, blinded to the treatment assignment of samples. This technology, previously described further in detail,^30^ was considered the gold standard for assessing three‐dimensional analysis of bone microstructure.

### 2.4 Biochemical analysis of serum parameters

Blood samples were allowed to clot, and the serum was collected by centrifugation. The levels of serum diamine oxidase (DAO) (Elabscience, China), D‐lactate (D‐LA) (Elabscience) and serum calcium (Nanjing Jiancheng Bio.) were measured by using commercial kits according to the manufacturer's protocols.

### 2.5 Real‐time PCR

The total RNA of the frozen colonic tissues and colonic organoids were extracted and quantified by using Eastep® Super Total RNA Extraction Kit (Promega, China). The homogenate of whole colonic tissues of each groups' rats were prepared by using an electric homogenizer. The reverse transcription (cDNA) was synthesized from 2 μg of total RNA using the GoScript™ Reverse Transcription System (Promega, China) according to the manufacturer's instructions. Quantitative Real‐time PCR was performed using 2 μl first‐strand cDNA with the GoScript® qPCR Master Mix (Promega), in a final volume of 20 μl. The following primers sequences were used: Cdh1, 5′‐TTGAGAATGAGGTCGGTGCC‐3′ (forward) and 5′‐CAGAATGCCCTCGTTGGTCT‐3′ (reverse); Ocln, 5′‐CTAAATTGGCATCCAGCCCAG‐3′ (forward) and 5′‐TCCTTTCCACTCGGGCTCA‐3′ (reverse); GADPH, 5’‐CAGTGCCAGCCTCGTCTCAT‐3′ (forward) and 5’‐AGGGGCCATCCACAGTCTTC‐3′ (reverse). The amplification condition was set at 40 cycles at 95°C for 15 s and 60°C for 30s using a LightCycler® 96 Instrument (Roche Life Science, Germany). The relative differences in expression of mRNA were measured by using 2^−ΔΔ^Ct method and normalized. The gene results for each rat's colon were expressed as log_10_‐transformed number of genome equivalent copies per ml by comparing the Ct values to the standard curves.

### 2.6 Western blotting

Total colon tissues and BMSCs cells were extracted by RIPA lysis buffer with 0.1% PMSF (Beyotime, China). Protein quantification was performed with the BCA assay kit (Beyotime). The homogenized protein samples were fractionated by 4%–20% pre‐cast gel (GenScript) at 120 V for 1.5 h, and transferred onto nitrocellulose filter (NC) membranes (Bio‐Rad Laboratories,USA) at 110–120 V for 1 h. The membranes were blocked in blocking buffer (Beyotime, China) for 15–30 min at room temperature, followed by an overnight incubation at 4°C with primary antibodies (Anti‐Occludin, #91131, 1:1000; Anti‐E‐cadherin, #14472, 1:1000; Anti‐LC3B, ab192890, 1:1000; Anti‐Caspase‐3, ab32351, 1:1000), and respective fluorescent secondary antibodies (Goat anti‐Mouse IgG H&L IRDye 680RD, ab216776, 1:5000; Goat anti‐Rabbit IgG H&L IRDye 800CW, ab216773, 1:5000) at 37°C for 1–2 h. Finally, the immunoreactive bands were visualized using Odyssey Clx fluorescence scanning system (LI‐COR Biosciences, USA). Equal protein loading was normalized with Anti‐β‐actin antibody.

### 2.7 16S rRNA sequencing and analysis

Total metagenomic DNA was extracted from 200 mg of each faecal specimen by using the E.Z.N.A.®Stool DNA Kit (Omega, Inc., USA) in accordance with manufacturer's instructions. High‐throughput sequencing of the V3‐V4 hypervariable region of the bacterial 16S rRNA gene was performed on NovaSeq PE250 platform according to the standard protocols with minor adjustments. PCR amplicons were generated with the primers 341F (5′‐CCTACGGGNGGCWGCAG‐3′) and 805R (5′‐GACTACHVGGGTATCTAATCC‐3′).^31^ The PCR conditions to amplify the prokaryotic 16S fragments consisted of an initial denaturation at 98°C for 30 s, 32 cycles of denaturation at 98°C for 10 s, annealing at 54°C for 30 s, and extension at 72°C for 45 s, and then final extension at 72°C for 10 min. The amplicon pools were prepared for sequencing. The size and the quantity of the amplicon library were assessed by Agilent 2100 Bioanalyzer (Agilent, USA) and the Library Quantification Kit for Illumina (Kapa Biosciences, USA), respectively.

After sequencing, the obtained reads were analysed using the QIIME2 pipeline for taxonomic classification. Then, by using DADA2, we obtained feature table and feature sequence. Alpha diversity was applied to measure the species diversity for samples through 2 indices, including Chao1 index and Good's Coverage. Beta diversity was visualized by principal coordinate analysis (PCoA) based on Unweighted Unifrac and analysis of similarities (ANOSIM Analysis). Blast was for sequence alignment, and the feature sequences were annotated with SILVA database for each representative sequence. Other diagrams were implemented using the R package (v3.5.2).

### 2.8 Serum and faecal metabolite extraction and LC/MS analysis

All the serum and faecal samples were thawed on ice and untargeted metabolic extraction was performed by LC/MS at Wuhan Metware Biotechnology Co, Ltd. Metabolic identification information was obtained by searching the laboratory's self‐built database and integrating the public database (Human Metabolome Database, HMDB) based on the exact masses of molecular ions. Through Variable Importance in Projection (VIP) filtering, which combined with Secondary spectrum score and difference multiples screening, differential metabolites were screened out. KEGG database was used for annotation of the different metabolites as described previously.^32^

### 2.9 Cell Culture

BMSCs (ATCC)were cultured in MEMα medium supplemented with 10% fetal bovine serum (FBS; Gibco), 1% antibiotic‐anti‐mycotic solution (Gibco) and 5 ng/ml bFGF (Gibco). Osteogenic differentiation media comprised the basal culture medium supplemented with 50 μM L‐ascorbic acid (Sigma), 10 mM β‐sodium glycerophosphate (Sigma) and 10 nM dexamethasone (Sigma). MC3T3 cells (Chinese Academy of Medical Sciences, China) were cultured in MEMα medium supplemented with 10% FBS (Gibco), 1% antibiotic‐anti‐mycotic solution (Gibco). RAW264.7 cells (RAW‐OCs, Chinese Academy of Medical Sciences)were cultured in DMEM medium supplemented with 10% FBS (Gibco) and 1% antibiotic‐anti‐mycotic solution (Gibco). The generation of colonic organoids from hiPSCs (CELLAPYBIO) involved a multistep technique whereby hiPSCs were directed to form definitive endoderm, hindgut structures and ultimate colonic organoids. hiPSCs were grown on matrigel (BD)‐coated six‐well plates in mTsSR1 medium (STEM CELL Technologies). All cells were maintained at 37°C with 5% CO_2_.

### 2.10 hiPSCs maintenance and colonic organoid differentiation

To induce definitive endoderm formation, hiPSCs were cultured on feeders to 70% confluence, and then plated at a density of 6000 clumps per well in a matrigel‐coated (24‐well plate). For accutase split cells, 10 μM Y27632 compound (Sigma) was added to the media for the first day. After the first day, media was changed to mTESR1 and cells were grown for an additional 24 hours. Cells were then treated with 100 ng/mL of Activin A for 3 days and treated with hindgut induction medium (RPMI 1640, 2 mM L‐glutamine, 2% decomplemented FBS, penicillin–streptomycin and 100 ng/mL Activin A) for 4 days with 500 ng/ml FGF4 (R&D) and 3 μM Chiron 99021 (Tocris) to induce formation of mid‐hindgut spheroids.

The mid‐hindgut spheroids were collected from 24‐well plates pooled and plated in Matrigel (BD) with a minimum of 30 spheroids plated per well. To generate human colonic organoids (HCOs), spheroids were overlayed with organoid basal medium (Advanced DMEM/F‐12, N2, B27, 15 mM HEPES, 2 mM L‐glutamine, penicillin–streptomycin) supplemented with 100 ng/ml EGF and 100 ng/ml BMP (Abcam) for initial 3 days. Media was changed at the fourth day with only EGF being maintained in the media for all patterning conditions. Perform a full‐medium change every 3–4 days one time. HCOs were replated in new Matrigel every 10 days at a density of 20–30 organoids per well.

### 2.11 Cell viability assay

Cells were seeded at a density of 2 × 10^4^ cells/well in 96‐well plates. After the cell confluence reached 80%, the cells were treated with 5‐HT from 10^−9^ to 10^−3^ M for 1 day and 3 days, respectively. The viability of cells were determined by the (3‐(4,5‐dimethylthiazol‐2‐yl‐)‐5‐(3‐carbxymethoxyphenyl)‐2‐(4‐sulfophenyl)‐2H‐tet‐razolium, innersalt) (MTS) assay. After medium removal, the cells were washed with PBS once, then 100 μl of MTS solution (Promega, China) consisting of 80 μl of MEMα and 20 μl of MTS solution were added to each well, and the cells were incubated for 3 h at 37°C. The absorption value (OD value) was measured at 490 nm using Multiskan Spectrum reader (Thermo Fisher). The results are expressed as the relative ratio to the control.

### 2.12 Alkaline phosphatase (ALP) staining and Alizarin red staining(ARS) assay

BMSCs were seeded at a density of 2 × 10^5^ cells/well in 6‐well plates. After the cell confluence reached 80%, the cells were treated with 10^−5^ M 5‐HT for 3 days. The experiment was then performed osteogenic induction for 14 days. Alkaline phosphatase (ALP) staining kit (Solarbio) was used to qualitatively detect the expression of ALP. Alizarin red staining (ARS) staining kit (Solarbio) was used to visualize the mineralization module formation of 5‐HT intervention. The cells were observed under a light microscope (Olympus) to evaluate the degree of osteogenic capability.

### 2.13 Immunofluorescence staining

The organoids were collected from 24‐well plates pooled and plated in Matrigel (BD, United States) with about 30 spheroids per well. After 100 mM ethanol intervention for 8 days, the spheroids were fixed in the plates with 10% formalin at room temperature for 60 min and washed three times with 1× PBS. They were then blocked in PBS with 5% horse serum and 0.3% Triton X‐100 for 1 h and incubated with primary antibodies (E‐cadherin, Cell Signalling Technology) overnight at 4°C, followed by Alexa‐488‐, Alexa‐Fluor‐555‐ conjugated Anti‐Mouse IgG secondary antibodies (1:1000; Beyotime).

### 2.14 Association analysis and statistical analysis

All data were expressed as mean values ± standard deviation (SD) for independent experiments. For comparison between two groups, a paired *t*‐test was performed. Statistical differences groups were analysed by one‐way ANOVA followed by the Tukey test. Spearman's correlation was used to show the relations between parameters of flora and metabolites. Statistical analysis was performed using R3.5.1 and SPSS 25.0 (IBM). *p* < 0.05 was considered statistically significant.

## 3 RESULT

### 3.1 Ethanol destroys the bone microstructure and leads to osteoporosis

**FIGURE 1:**
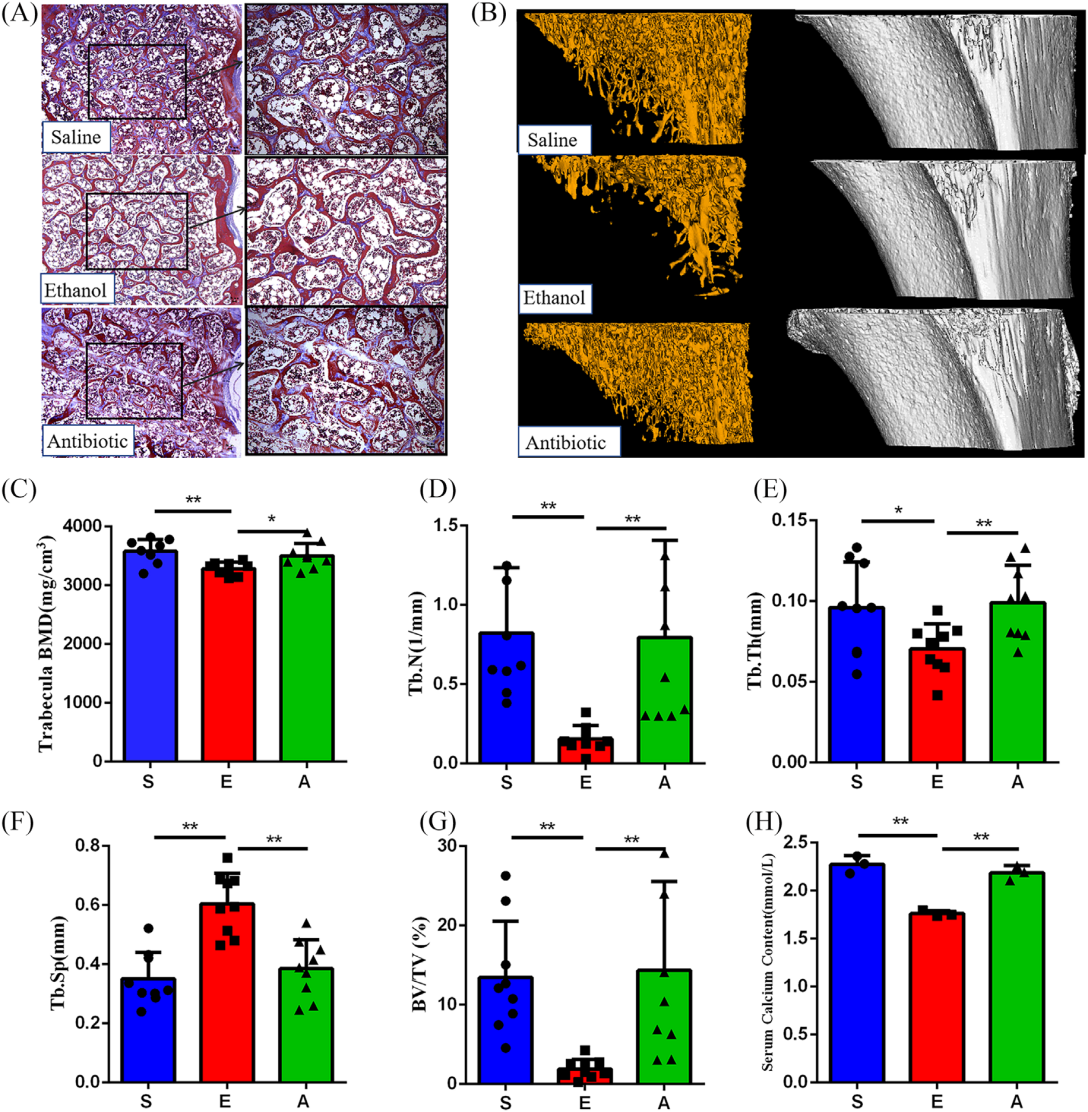
Effects of ethanol and antibiotics on bone metabolism. (A) Masson staining of the fifth lumbar vertebra. The images consist of representative photos in 100× magnification (left) and in 200× magnification (right) (*n* = 3). (B) Representative micro‐CT images of cancellous bone. (C–G) Changes in different trabecular micro‐architecture parameters (*n* = 9). (H) Comparison of the serum calcium levels among the three groups (*n* = 3). Values represent as bar graph with mean ± standard deviation (SD). *p < 0.05, **p < 0.01

To investigate the effect of ethanol on bone, we determined the microstructure of trabeculae by micro‐CT and Masson staining. As shown in the Masson staining samples, the trabecular bone showed thinner, less in quantity, lower in density and sparser arrangement in the ethanol group (Figure 1A). In addition, the staining results showed that there were less osteoid tissues around the ethanol group than the control group. Otherwise, the collagen located at the trabeculae, which represented the bone matrix content, was obviously reduced in the ethanol group. To quantitatively confirm the degree of change in the content of cancellous bone, Micro‐CT was used to measure the bone density and bone volume. The ethanol group was manifested a deteriorated bone architecture by the three‐dimensional reconstruction of micro‐CT. Trabecular bone mineral density (BMD) and bone volume fraction (BV/TV) decreased significantly in the ethanol group (*p* < 0.01, Figure 1C,G), and a similar trend was found in trabecular number (Tb.N) (*p* < 0.01) and trabecular thickness (Tb.Th) (*p* < 0.05, Figure 1D,E). In contrast, trabecular separation (Tb.Sp) increased significantly in ethanol group (*p* < 0.01, Figure 1F). Otherwise, the decreasing calcium in serum also reflected an imbalance in bone metabolism (*p* < 0.01; Figure 1H), even though the phosphate levels is not change significantly. Taken together, these results verified that the animal model of osteoporosis successfully established, which caused by chronic heavy drinking. Meanwhile, the antibiotic group did not cause bone quality changes.

### 3.2 Ethanol impairs the gut epithelial barrier integrity

**FIGURE 2:**
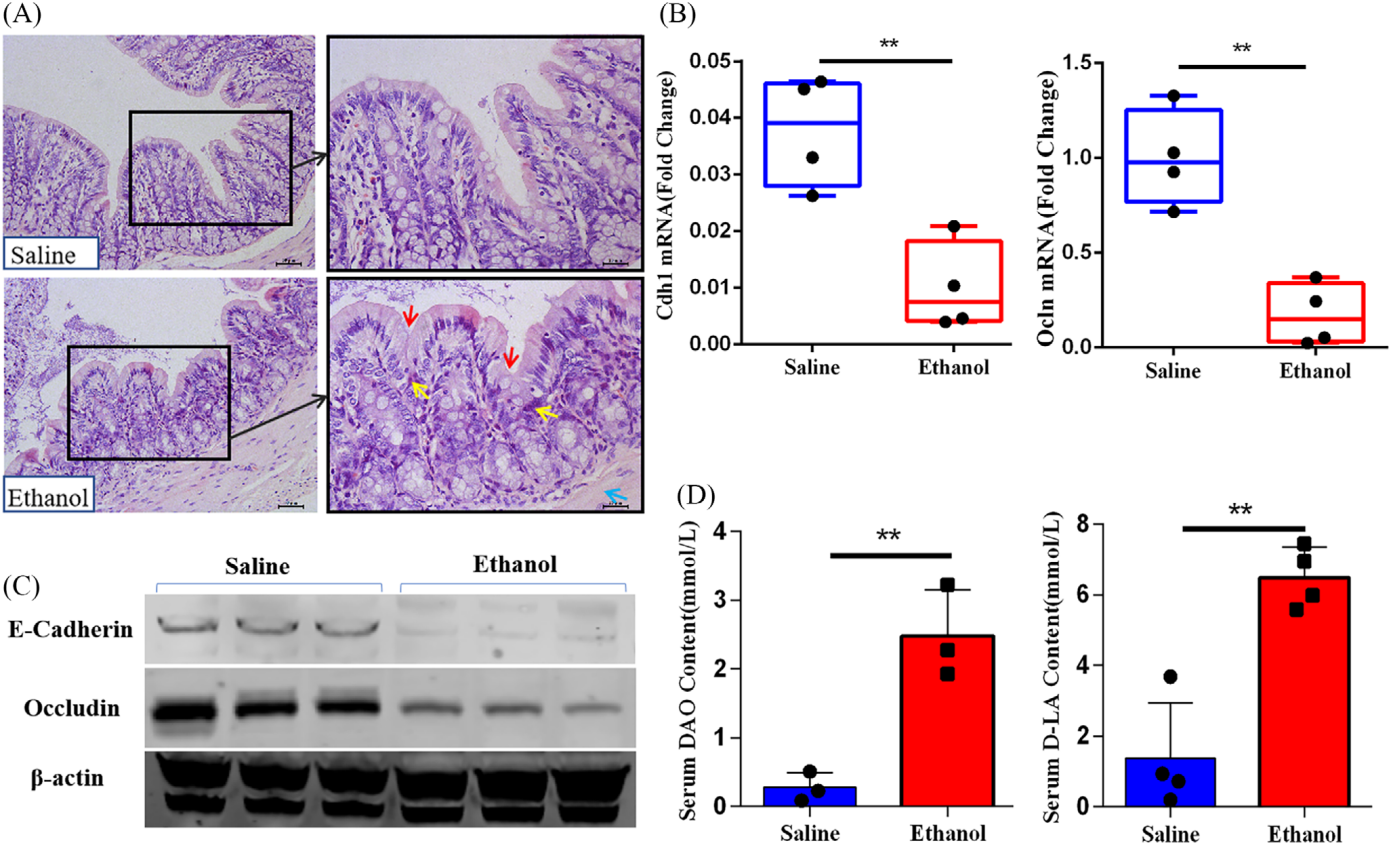
Representative images of gut epithelial barrier function test between ethanol‐induced osteoporosis and control. (A) Colon histopathology. In the haematoxylin and eosin staining images, red arrows reflect the decrease of goblet cells, yellow arrows reflect the breach of immune cells into the mucosal compartment (i.e. increased cellularity), and the blue arrow highlights the muscularis externa. The images consist of representative photos in 200× magnification (left) and in 400× magnification (right) (*n* = 3).The genes (B, *n* = 4) and proteins (C, *n* = 3) expression levels of E‐cadherin and Occludin from different groups. The biomarkers of gut leakage, DAO (D) and D‐LA (E) in serum, are detected (*n* = 3). Values represent as bar graph or box plot with mean ± standard deviation (SD). *p < 0.05, **p < 0.01

In order to observe the pathological changes of colon tissues treated with ethanol, the histological sections were evaluated by H&E‐staining. The colonic tissue of Ethanol group showed obvious infiltration of inflammatory cells, decrease of goblet cells and the muscularis externa were widened (Figure 2A). We determined the relative expression of tight junction genes, including Ocln and Cdh1, and found significantly decreased expression in the E group compared with the S group (*p* < 0.01, Figure 2B). In Western Blot, we also saw that the expression levels of connexin, E‐cadherin and Occludin in the colon tissue of E group were lower than those of the S groups (Figure 2C). Furthermore, gut leakage biomarkers in rat serum were also determined. Our results showed that serum diamine oxidase (DAO) and D‐lactose (D‐LA) were significantly enriched in the E group (*p* < 0.01, Figure 2D,E). These results demonstrated that the function of the gut epithelial barrier was impaired by ethanol, which might cause intestinal microorganisms or their toxins leaking into the body.

### 3.3 Ethanol alters the balance of intestinal bacterial diversity

**FIGURE 3:**
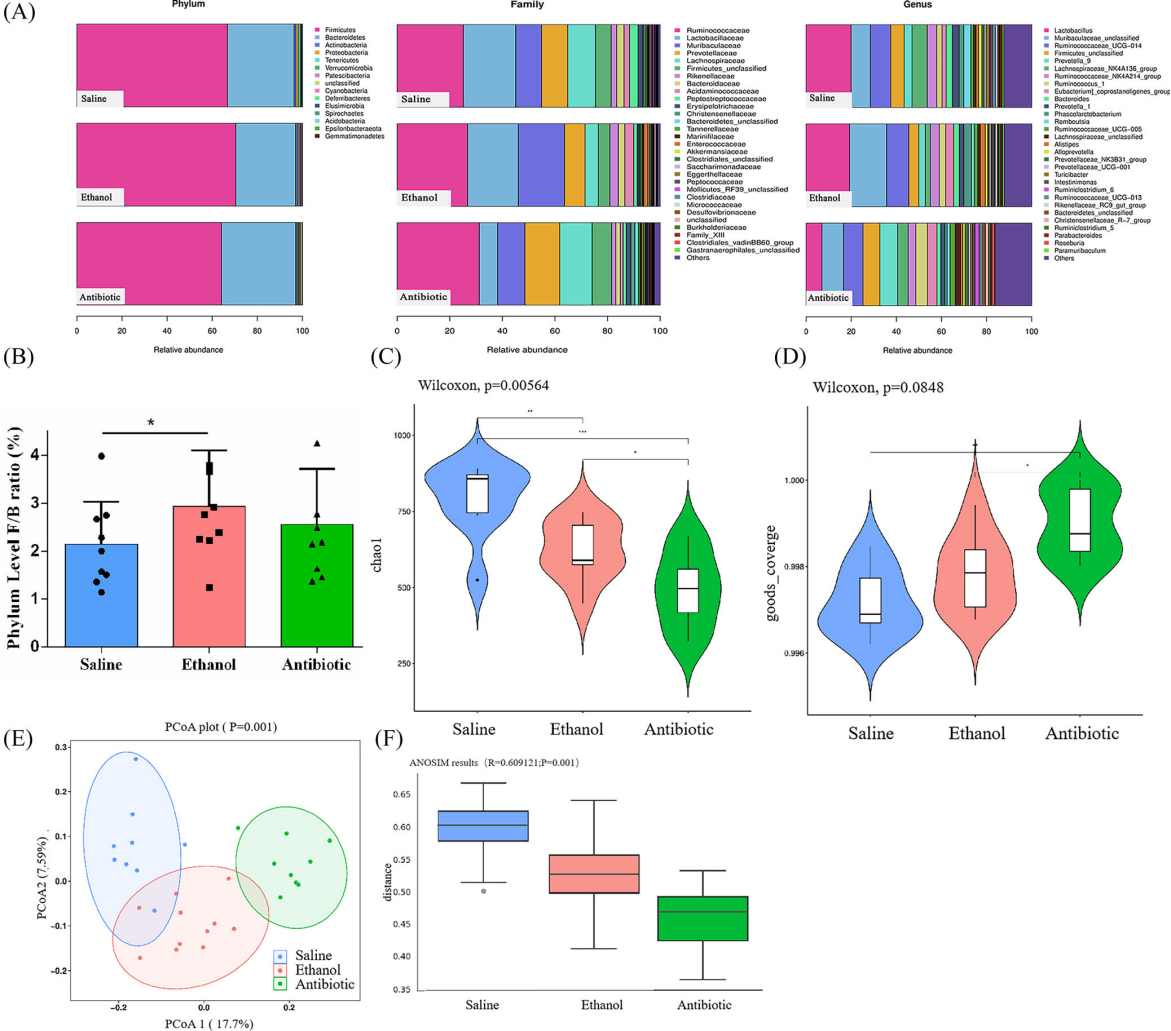
Effect of ethanol and antibiotic gavaged on gut‐microbiota composition (*n* = 9). (A) Annotation of phylum, family and genus level for the three groups. (B) Firmicutes/Bacteroidetes ratio (F/B ratio) at phylum level. Alpha diversity analysis, Chao1 (C) and goods coverage (D) represent the difference in diversity within the three groups. Beta diversity analysis, PCoA plot (E) and ANOSIM plot(F), represent the difference in diversity among the three groups. * *p* < 0.05, ** *p* < 0.01

The 16S rRNA gene sequencing data were obtained from the three group, 27 stool samples, with a mean of 66,965 ± 11,179 sequences per specimen. A total of 1,808,057 high‐quality reads were obtained after noise dropout and error correction. According to the results of the Kruskal‐Wallis rank sum test and linear discriminant analysis, the phylum abundance of *Proteobacteria*, *Tenericutes*, *Actinobacteria* and *Firmicutes* was increased and the abundance of *Bacteroidetes* was decreased in the E group and A group as compared to the S group. Among them, the change level of *Firmicutes*/*Bacteroidetes* ratio in E group was significantly (*p* < 0.05, Figure 3A,B).

To check whether the sequencing data was sufficiently, rarefaction analysis (Chao1 and Good's Coverage index) was performed for these samples. Chao1 estimator represented the level of bacterial community richness and evenness, which was significantly lower in the E group and A group than the S group (Figure 3C). Good's Coverage index was significantly higher in the A group than the S group and E group (Figure 3D). Meanwhile, there was no statistic difference in terms of the Good's Coverage index between E group and S group (eventhough the average value of S group was slightly lower than that of E group). The value in each group was near saturation (saturation value = 1), which suggested that the sequencing data was sufficiently robust and few new species was undetected. We further performed Unweighted UniFrac‐based principal coordinates analysis (PCoA) and Analysis of similarities (ANOSIM) based on Feature abundances and found significant differences among the three groups. In detail, PCoA revealed a distinct clustering of the GM composition in each group (Figure 3E). ANOSIM showed that the E group was significantly different from S and A groups (Figure 3F), suggesting that ethanol‐induced osteoporosis as well as GM dysbiosis.

**FIGURE 4:**
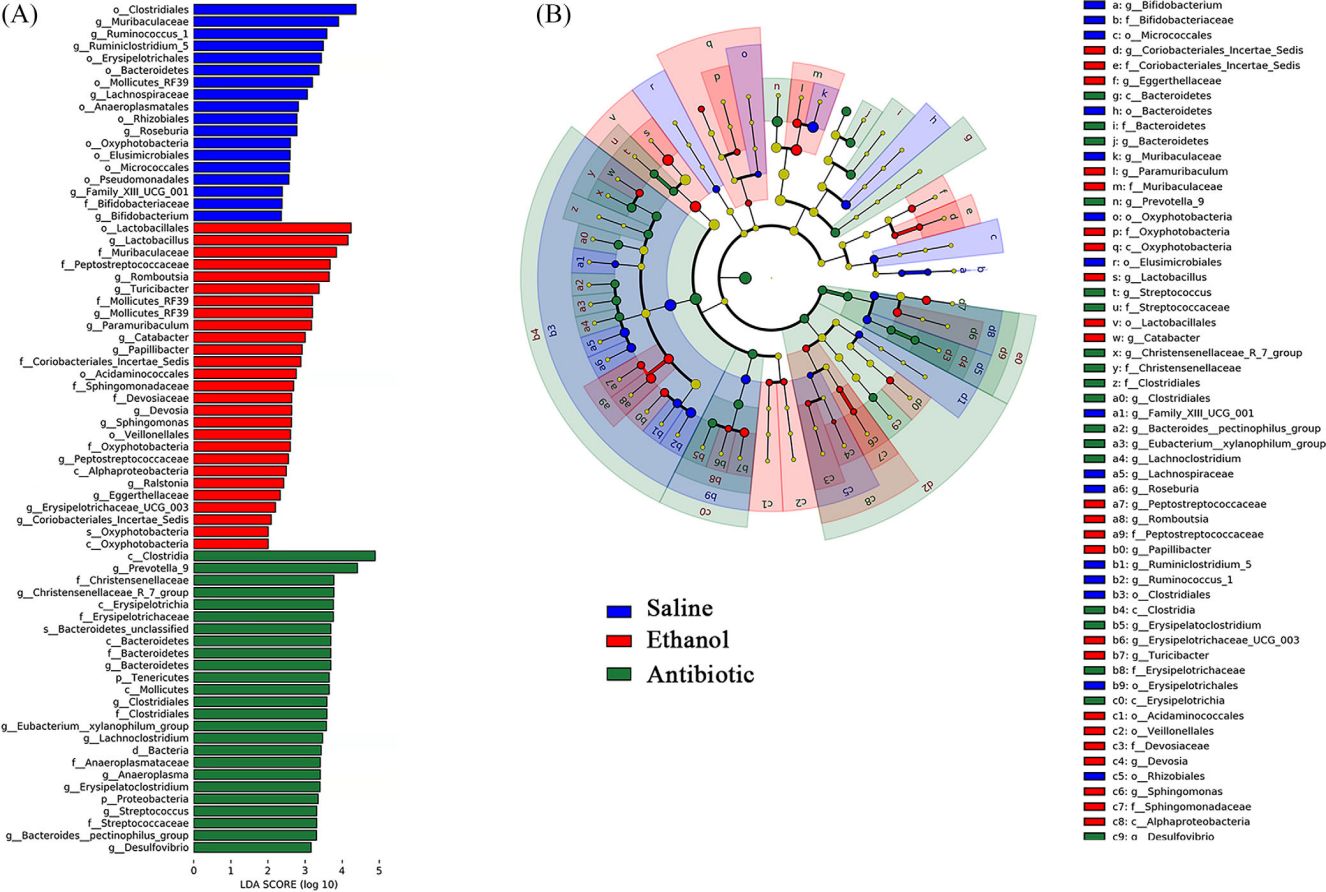
Discriminativ taxa among the three group (*n* = 9). (A)LDA (Linear discriminant analysis) image shows the differential bacterial genus in each groups. (B) The cladogram represents biomarkers in different groups (Saline in blue, Ethanol in red and Antibiotic in green). The size of point shows the negative logarithms (base 10) of *p*‐value. The bigger size of point shows more significant (lower *p*‐value)

At the genus level, the relative abundances of *Catabacter*, *Peptostreptococcaceae*, *Lactobacillus*, *Turicibacter*, *Erysipelotrichaceae_UCG‐003* and *Romboutsia* belonging to the *Firmicutes* phylum, were significantly higher in the E group than the other two groups (Figure 4A,B). Furthermore, the relative abundances of *Bifidobacterium*, *Lachnospiraceae*, *Muribaculaceae* and *Roseburia* were significantly higher in the S group (Figures 3A and 4A). These species‐specific changes may be highlight candidates in the pathogenesis of EIO.

### 3.4 Serum and faecal metabolism profiles in ethanol‐induced osteoporosis

**FIGURE 5:**
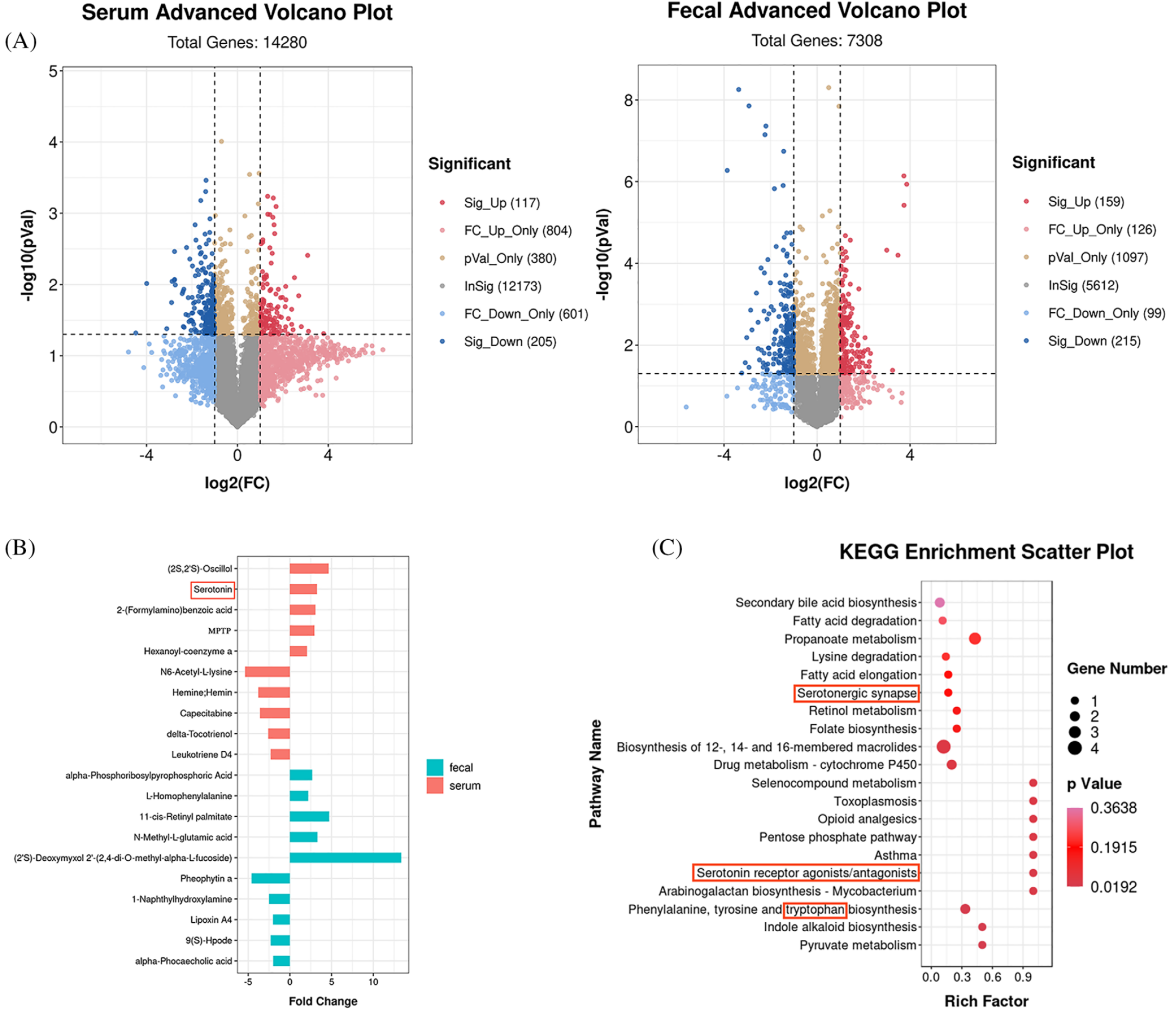
Faecal and Serum Metabolomic Signatures in osteoporosis (*n* = 9). (A) Volcano plot represents the difference changes in serum and faecal metabolites after ethanol‐gavaged. (B) Removing the metabolites that coexisting both in the Ethanol group and Antibiotic group, the top 5 and bottom 5 differential metabolites caused by ethanol were screened out. (C) Enrichment analysis for differential metabolite pathways. The abscissa represents the Rich factor corresponding to each pathway, the ordinate represents the pathway name, and the colour of the point represents *p*‐value. The colour of the points represents the significance of the enrichment. The size of the dot represents the number of different metabolites enriched

The serum and faecal metabolic profiles were analysed by LC/MS. Differences metabolite profiles of the three groups were revealed by OPLS‐DA. A total of 322 and 374 differential metabolites were identified in serum and faeces, respectively, between the S group and E group (Figure 5A). These metabolite features were largely distinguished subjects with osteoporosis from those non‐osteoporosis, which indicated of broad metabolic differences between the two groups. Compared with the S and A group, ethanol‐gavaged rats had higher concentrations of (2S,2′S)‐Oscillol, 2‐(Formylamino) benzoic acid, Serotonin (5‐HT), 1‐Methyl‐4‐phenyl‐1,2,3,6‐tetrahydropyridine (MPTP), and hexanoyl‐coenzyme A in serum and alpha‐Phosphoribosylpyrophosphoric acid, L‐Homophenylalanine, (2′S)‐Deoxymyxol 2′‐(2,4‐di‐O‐methyl‐alpha‐L‐fucoside), N‐Methyl‐L‐glutamic acid, and 11‐cis‐Retinyl palmitate in faecal. In addition, N6‐Acetyl‐L‐lysine, hemine, delta‐Tocotrienol, Leukotriene D4 and Capecitabine in serum and pheophytin a, 1‐Naphthylhydroxylamine, 9(S)‐Hpode, Lipoxin A4 and alpha‐Phocaecholic acid in faecal were significantly declined in E group (Figure 5B). Further metabolite enrichment analysis indicated that ethanol had significant impact on multiple biological metabolism pathways, such as amino acid metabolism, bile acid biosynthesis, purine and pyrimidine metabolism, biosynthesis of alkaloids, fatty acid metabolism and so on (Figure 5C). For another, antibiotic group's metabolic profiles were more broadly distributed, but the difference did not caused the bone mass variation.

**FIGURE 6:**
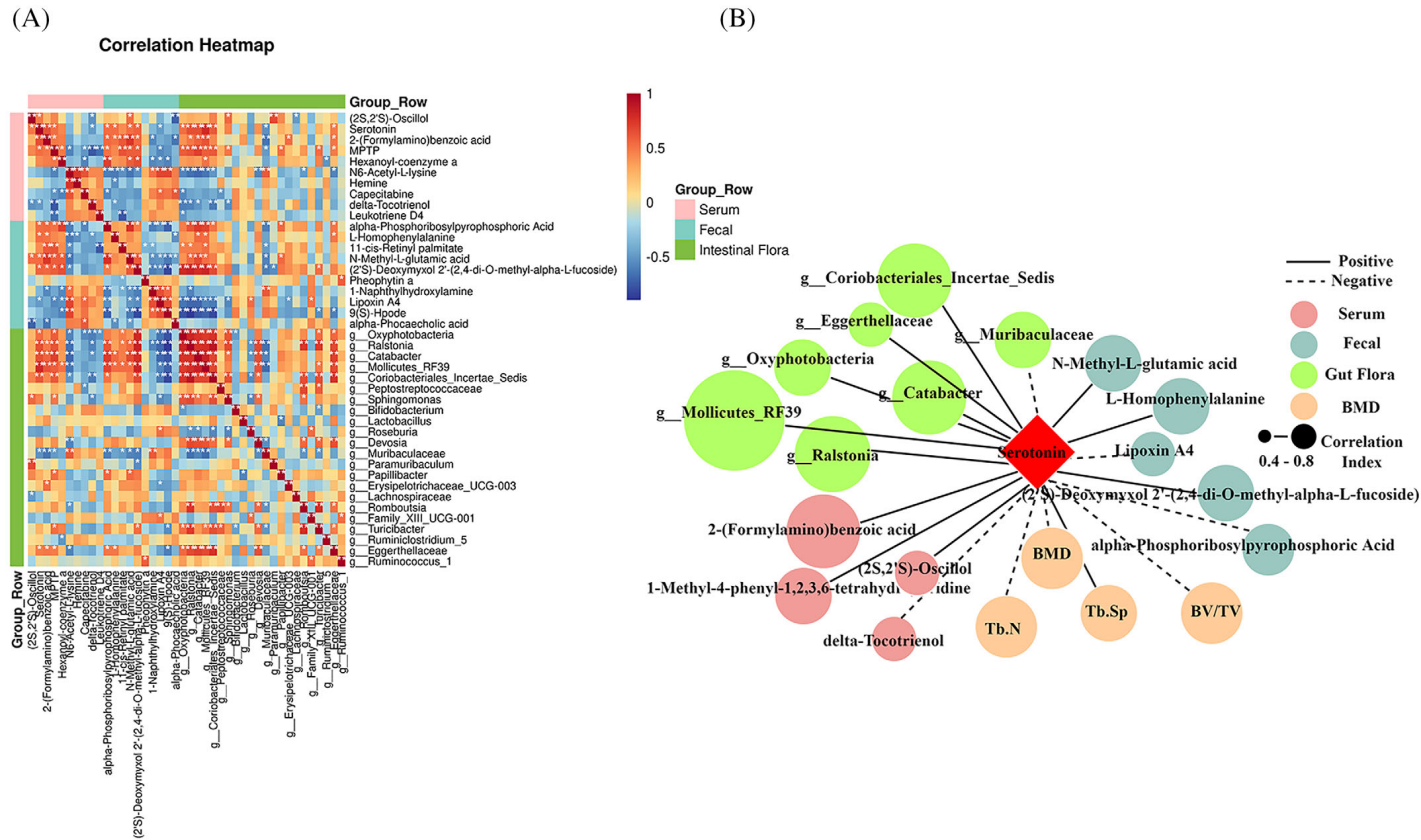
Correlation analysis diagram. (A) Heatmap of correlations between the top five and bottom five of serum metabolites and faecal metabolites with differential GM. Red indicates a strong positive correlation, and blue indicates a strong negative correlation. (B) Serum, faecal metabolites, GM, and bone metabolism parameters associated with 5‐HT. Solid lines indicate positive correlations, while dashed lines indicate negative correlations. *p < 0.05, **p < 0.01.

The relationships among the different bacteria, different metabolites in serum and faecal were examined by correlation analysis (Spearman). The study found that (2S,2′S)‐Oscillol, MPTP and 2‐(Formylamino) benzoic acid had significant positive correlations with 5‐HT in the serum (Figure 6A,B), while MPTP and Hexanoyl‐coenzyme A had significant negative correlation with Capecitabine. At the same time, 5‐HT and MPTP were positively correlated with *g_Oxyphotobacteria*, which were significantly negatively correlated with Capecitabine. We also found that delta‐Tocotrienol was negatively correlated with many high levels of serum metabolites, including (2S,2′S)‐Oscillol, MPTP and 5‐HT. It had already been reported that delta‐Tocotrienol was associated with osteoblast differentiation and promoted alkaline phosphatase synthesis.^32^ Interestingly, delta‐Tocotrienol also has significant negative correlations with *g_Oxyphotobacteria*. In the faecal metabolism, osteoporosis‐reduced Lipoxin A4 correlated negatively with *g_Oxyphotobacteria* and *g_Ralstonia*, which had reported that it could inhibit the activity of osteoclasts and reduced the bone loss caused by ovariectomized rats.^33^ In addition, we found that osteoporosis‐enriched N‐Methyl‐L‐glutamic acid and alpha‐Phosphoribosylpyrophosphoric acid in the faecal were positively associated with the same five genera. These five genera included *g_Muribaculaceae*, *g_Ralstonia*, *g_Catabacter*, *g_Mollicutes_RF39* and *g_Papillibacter* (Figure 6A). Next, we chose serotonin (5‐HT), which had been reported in the literature, as the core target to observe whether it affected the balance of bone metabolism.^34^ Through correlation analysis, we found that four kinds of serum metabolites, five kinds of faecal metabolites and seven kinds of different bacterial genera had a clear correlation with the change levels of 5‐HT (Figure 6B). Altogether, these results indicated that the distinguishing metabolites were closely related to GM variation and the distinguished metabolites and GM were related to osteoporosis, even though it remains to be explored whether these metabolites are directly produced by the intestinal bacteria.

### 3.5 The effect of ethanol on the colonic organoid

**FIGURE 7:**
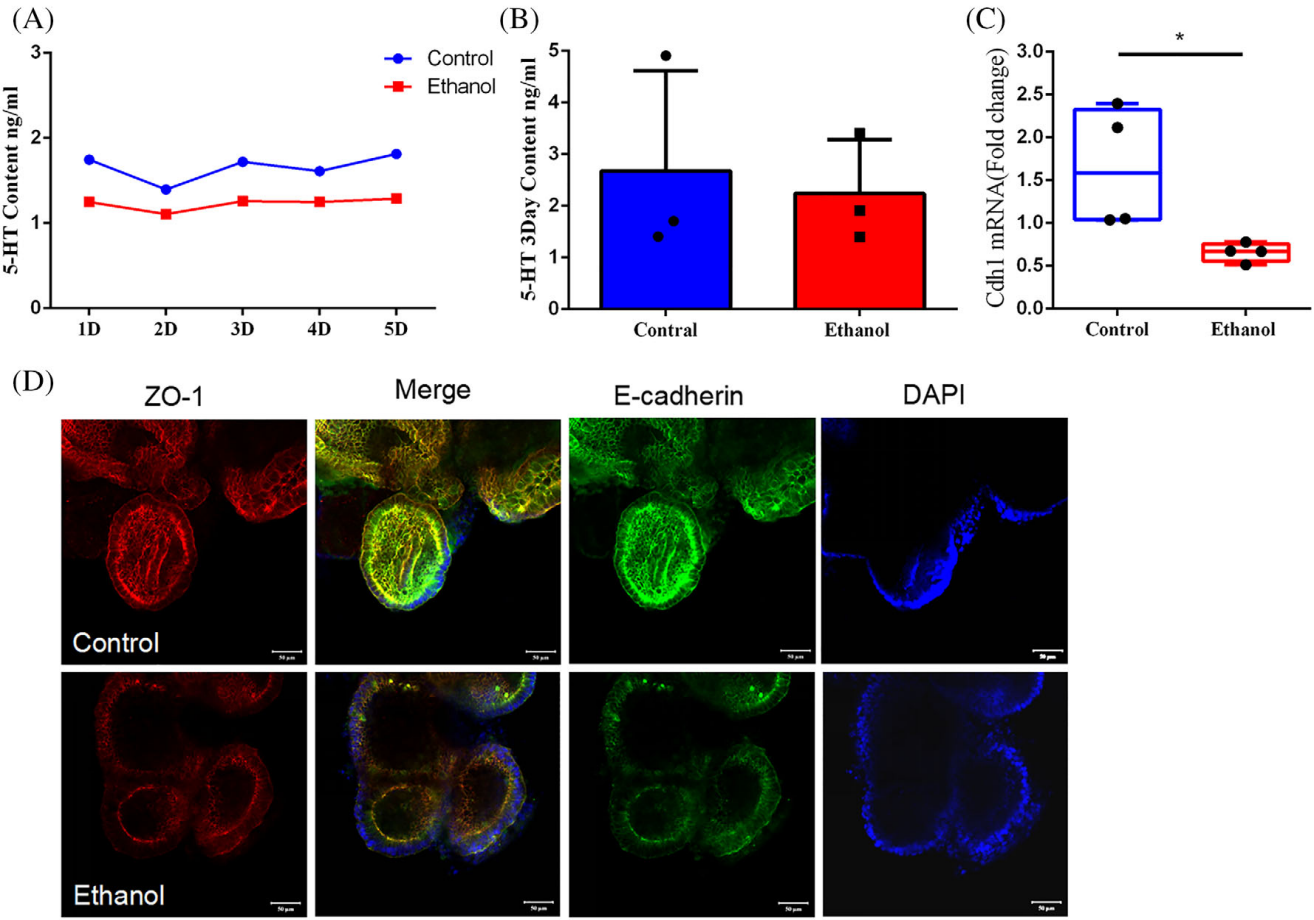
The effects of ethanol on 5‐HT content from colon organoids. The 5‐HT content of colon organoids supernatant are detected by UPLC for 1–5 days (A) and for 3 days accumulation (B) (*n* = 3); Immunofluorescence images reveal the E‐cadherin and ZO‐1 protein expression level (C), and Cdh1 gene expression level is detected by q‐PCR (D) (*n* = 4). *p < 0.05, **p < 0.01

In vitro, we successfully cultured and induced iPSCs cells into colonic organoids. In the organoid supernatant test for five consecutive days, we found that the release content of 5‐HT by colonic organoids in the ethanol group was slightly lower than that in the control group, while there was no statistical significance (Figure 7A). The accumulation content of 5‐HT in the organoid supernatant for 3 days was still no significant difference between the two groups (Figure 7B). We also found the levels of the Cdh1 genes depression (p < 0.05, Figure 7C). Through immunofluorescence detection, we found that the expression of E‐cadherin and ZO‐1 protein on the surface of colon organoids decreased after ethanol intervention (Figure 7D).

### 3.6 Serotonin cause osteogenesis inhibition but not osteoclast enhancement

**FIGURE 8:**
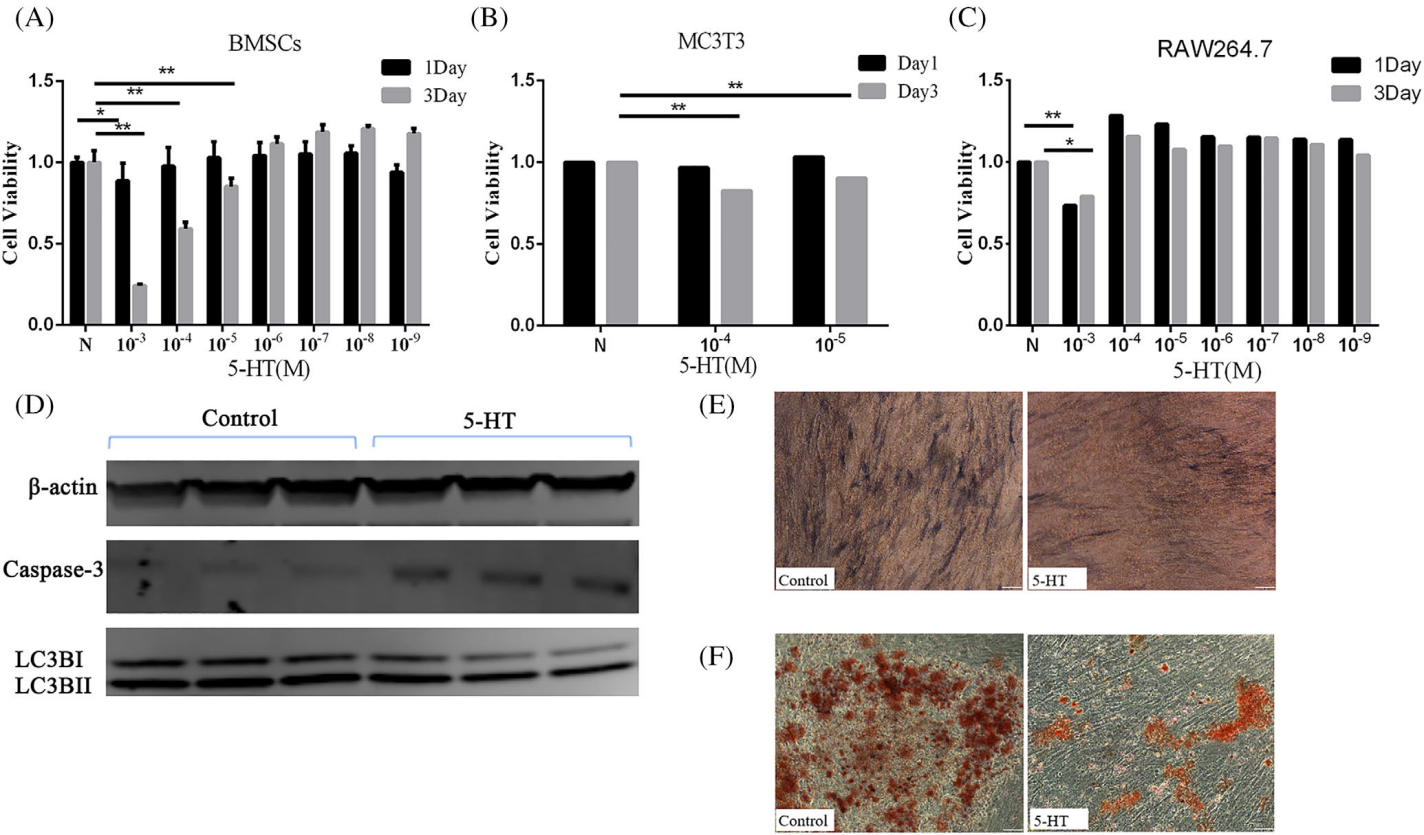
Inhibitory effect of 5‐HT on osteogenesis. Cell viability experiments show the effect of 5‐HT on BMSCs (A), MC3T3 cells (B) and RAW264.7 cells (C) (*n* = 3). The proteins (D) expression levels of Caspase‐3 and LC3BI/II from different groups (*n* = 3).Representative ALP staining (E) and Alizarin red staining (F) images of BMSCs (*n* = 3). The representative images are in 100× magnification. Values represent as bar graph with mean ± standard deviation (SD). *p < 0.05, **p < 0.01

We used different concentrations of serotonin (5‐HT) to intervene the bone mesenchymal stem cells (BMSCs), MC3T3 cells and RAW264.7 cells for cell viability testing (MTS). The data showed that, in BMSCs, 1 mM 5‐HT intervention for 1 day could affect cell viability, while 10 μM and 100 μM 5‐HT could affect cell viability during 3 Days of intervention (Figure 8A). The same trend could be seen in MC3T3 cells under 5‐HT intervention (Figure 8B). However, for 1 day and 3 days intervention experiments on RAW264.7 cells, we only saw that 1 mM 5‐HT affects cell viability (Figure 8C). The lower concentration of 5‐HT promoted the viability of osteoclasts instead (no statistical difference). After 5‐HT treatment, the apoptosis marker, Caspase‐3, was significantly up‐rugulated, while the expression of autophagy marker LC3B I/II was not significantly change (Figure 8D). Otherwise, we also saw that 5‐HT had an inhibitory effect on the bone formation and mineralization ability of BMSCs (Figure 8E, F).

## 4 DISCUSSION

There is solid evidence that chronic heavy ethanol consumption has detrimental effects on bone health and increases the risk of osteoporosis.^35^ Although heavy drinking negatively impacts bone formation, the underlying mechanisms by which ethanol affects bone turnover are poorly understood. Alcoholic injury is multi‐systemic, predominantly the alcoholic liver disease (ALD). In ALD patients, alcohol impairs the gut epithelial barrier and aggravates ALD through activation of inflammation induced by bacterial endotoxin and lipopolysaccharide. Moreover, the changes in gut microbiota composition occur before ALD development. These results demonstrate that alterations in the gut microbiome are recognized as a major factor in the ALD progression.^36^ Studies in the amount and diversity of bacterial populations of patients with osteoporosis indicate that osteoporotic adults appear to have reduced diversity of GM, which reveals that GM is also a central regulator of bone homeostasis and the pathogenesis of osteoporosis,^37^, ^38^ in what is now being called the gut–bone axis. However, the mechanism underlying this connection how ethanol affects bone remodelling via GM composition is still somewhat enigmatic.

To validate the correlation between GM dysbiosis and osteoporosis in this study, GM composition by 16S rRNA gene seq and bone density by micro‐CT are tested in rats with chronic ethanol intake. Meanwhile, antibiotics are applied in rats for 16 weeks as a GM dysbiosis matched group, which has been proven to reduce taxonomic richness and diversity and influence the abundance of bacterial taxa in faeces.^39^ Previous studies have shown that the bone strength is not affected by long‐term low‐glycemic diet containing antibiotics, but GM is significantly changed.^40^ Here, we report that the bacterial community richness and evenness are lower in the faecal samples from ethanol and antibiotics treated rats using the Chao1 estimator. Moreover, the differences in bacterial community structure are further investigated by PCoA and ANOSIM, indicating the differences in the structure and composition of GM among groups, which highlights the induction of GM dysbiosis in chronic heavy ethanol consumption. To explore the differences in detail, the results from the Kruskal‐Wallis rank sum test and linear discriminant analysis reveal a significantly higher abundance of *Proteobacteria*, *Tenericutes*, *Actinobacteria*, *Firmicutes*, and a lower abundance of *Bacteroidetes* at the phylum level in the faecal samples from rats treated with ethanol and antibiotic compared with those from the rats treated with saline, indicating the occurrence of GM dysbiosis. *Firmicutes* and *Bacteroidetes* are the main phylum of the GM that can be identified in each group, accounting for about 96% of the total microbiome. The variation of *Firmicutes* to *Bacteroidetes* (F/B) ratio has been proved to be closely related to osteoporosis in individuals, however, the results are still controversial.^41^, ^42^ In our results, the F/B ratio in ethanol‐treated rats increases to 2.94, higher than the ratios from the other two groups, 2.03 in the saline group and 2.55 in the antibiotics group. Further analysis for the composition of *Firmicutes* phylum at the genus level, *Catabacter*, *Peptostreptococcaceae*, *Lactobacillus*, *Romboutsia*, *Erysipelotrichaceae_UCG‐003*, *Papillibacter* and *Turicibacter*, particularly enrich in ethanol‐treated rats. In addition, the populations of *Bifidobacterium* and *Ruminococcus_1* also reduce in the GM from ethanol‐treated rats. *Bifidobacterium* is reported to reduce the concentration of inflammatory factors to prevent osteoclast activation and bone resorption.^43^, ^44^ *Ruminococcus* protect intestinal mucus from degradation against the entry of harmful substances into the blood.^45^ Consistent with decreased *Ruminococcus*, we find that the gut epithelial barrier shows partial impairments in the ethanol‐treated rats. Together, our findings demonstrate the heterogeneity in the GM dysbiosis induced by ethanol and antibiotics. Extrapolating the concept that GM dysbiosis influences bone healthy^46^ leads us to wonder whether ethanol and antibiotics have similar roles in osteoporosis. Notably, the results from micro‐CT and Masson staining demonstrate that ethanol destroys the bone microstructure and inhibits the bone turnover, whereas antibiotics exert no effects on the bone density and structure. These results reveal a specific pattern of ethanol‐related changes in GM that may impact bone remodelling, especially in the bone microstructure. Species‐specific changes of GM are related to the integrity of mucosal barrier and immune/cytokine signalling, and may jointly contribute to ethanol‐dependent susceptibility to osteoporosis.

GM plays pivotal roles in the metabolism of dietary components and specific bacteria have been involved in different processes, therefore, GM dysbiosis not only impacts the metabolism of dietary components but also alters some host‐generated substances.^47^ Having demonstrated that impairments of gut epithelial barriers occur in a rat model of ethanol‐induced osteoporosis, we seek to identify differentially endogenous metabolites that could be specifically targeted as biomarkers for bone remodelling. A total of 322 and 374 differential metabolites are identified in the serum and faeces of the ethanol‐treated group, respectively. Compared with the saline group, the differential enriched metabolites identified in both ethanol and antibiotics are excluded due to the lack of phenotypic effects of antibiotics in osteoporosis. Finally, the top five and bottom five of serum metabolites and faecal metabolites are selected for analysis, respectively. These metabolites are participated in multiple biological pathways by KEGG analysis, including multiple amino acids, bile acid biosynthesis, purine and pyrimidine metabolism, and alkaloids and fatty acid metabolism. The metabolic pathways enrichment analysis shows that tryptophan, as the hub of metabolite, participates in multiple pathways (Figure 5C). Consistent with this, we also see elevated serum levels of tryptophan downstream metabolites serotonin and 2‐(Formylamino) benzoic acid in our experiments. Furthermore, Spearman correlation analysis indicate there is a positive correlation between the serum serotonin and the six kinds of different bacterial genera. To further compare differential metabolites between faeces and serum, unexpectedly, no same metabolites are identified and singled out. This result is inconsistent with the concept that faecal metabolites may secret into blood due to the damage of gut epithelial barriers.^48^ This result may be caused by the different metabolic rates of metabolites in blood and faeces.

Notably, gastrointestinal tract is the main site of tryptophan metabolism. The latest evidence of subtle interactions between serotonin and bone remodelling is still controversial.^49^ It is reported that the gut‐derived serotonin (g5HT) derived from enterochromaffin cells is partially modulated by GM and has the potential in decreasing osteoblast proliferation.^50^ Meanwhile, circulating elevated levels of 5‐HT are also reported in DSS‐induced mouse colitis and lead to significant deficits in trabecular bone mass.^51^ Whereas, Cui et al. show no bone phenotype variation in both knockouts of 5HT receptor 1 and decrease of circulating 5‐HT.^52^ Considering the increased serum 5‐HT and the decreased bone identity, we hypothesize that 5‐HT plays a role in ethanol‐induced bone remodelling. Firstly, we need to answer is whether the elevated serum level of 5‐HT induced by alcoholic is derived from enterochromaffin cells and which factors stimulate the cells to secret 5‐HT. To test this idea, we treat colon organoids with ethanol and measure the level of 5‐HT in the supernatants. Colon organoids differentiated from iPSC is a more physiologic modality to mimic multicellular organs as compared to the traditional single cell two‐dimensional culture.^53^ The result reveals that continuous ethanol stimulation does not elevate the secretion of 5‐HT, which suggests that ethanol has no direct effects on the 5‐HT secretion stimulation. Although it is not sufficient to make conclusions that ethanol modulates serum 5‐HT by GM, the evidence is pointing toward the GM dysbiosis caused by ethanol. Notably, in agreement with the impairments of gut epithelial barriers in vivo, the expression of connection protein, E‐cadherin and ZO‐1, declines upon the ethanol exposure in intestinal organoids.

Next, we seek to determine whether the 5‐HT effects on bone formation arise in osteogenic precursor cells. The results from the cell viability assay reveal that 5‐HT reduces the cell viability of bone mesenchymal stem cells (BMSCs) and osteoprogenitor MC3T3 cells in a dose‐dependent way. Interestingly, the same dose of serotonin does not affect the cell viability of the osteoclast precursor cells, RAW264.7. These results suggest that the elevated 5‐HT may disrupt the dynamic balance between osteo‐formation and osteo‐resorption during bone metabolism and ultimately lead to osteoporosis. To verify how the proliferation inhibition of 5‐HT in BMSCs arises, we examine the role of apoptosis pathway in the cell damage. Our results show that the activation of casepase3 in BMSCs is significantly increased after 5‐HT treatment. Furthermore, serum 5‐HT significantly inhibits the osteogenic activity and the bone mineralization of BMSCs during osteogenic differentiation.

## 5 CONCLUSIONS

In conclusion, our study demonstrates that chronic heavy ethanol consumption causes osteoporosis and GM dysbiosis in rats, indicating that the regulation of the gut‐bone axis might contribute to the ethanol‐induced bone loss. Conjoint analysis of the genetic profiles of GM and metabolic phenotypes in serum and faeces reveals the increased serum endogenous metabolite, 5‐HT, were significantly correlated with the GM dysbiosis, the changes of seven differential gut microbiota, including *g_catabacter*, *g_Mollicutes_RF39*, *g_Ralstonia* and so on. Further, we find that the elevated 5‐HT level impairs the bone formation in vitro, which reaffirms that the GM dysbiosis might be an indirect pathogenic factor to disrupt the bone formation in the ethanol‐induced osteoporosis. Although the specific subtypes of GM that impairs bone formation are unknown for lack of a proof‐of‐concept study, these results indicate that maintaining the GM haemostasis might be a viable treatment strategy for serotonin‐related gut‐bone axis disorders.

## AUTHOR CONTRIBUTIONS

Zhao Liu and Yunlong Hou designed the studies and wrote the manuscript with input from all authors. Xiaodong Li and Hang Lv performed the methodology and investigation. Yiwei Shen and Wenwen Cui performed the animal experiment. Zhao Liu and Xin Zhang performed the histological analysis on bone and colon tissues and analysed the data. Wenyan Li and Yuanyuan Hao participated in the cultivation and experiment of organoids. Xiaofeng Zhang and Xilin Xu supervised all studies and the drafting and editing of the manuscript.

## CONFLICT OF INTEREST

The authors declare no conflict of interest.

## Data Availability

The gut microbiota datasets used and analyzed during this study are available at the NCBI Sequence Read Archive (SRA), under BioProject PRJNA808654.
